# Hauntological dimensions of heart transplantation: the onto-epistemologies of deceased donation

**DOI:** 10.1136/medhum-2020-011982

**Published:** 2021-02-26

**Authors:** Margrit Shildrick

**Affiliations:** ERG, Stockholms Universitet, Stockholm, Sweden

**Keywords:** medical humanities, philosophy of medicine/health care, cardiology, cultural studies

## Abstract

The practice of human organ transplantation studies is shot through with questions concerning the concepts of selfhood and identity that continually reach out towards transmigration, displacement and haunting. In particular, heart transplantation is the site at which the parameters of human life and death are tested to their limits, not simply for the recipient but for the donor too. In conventional biomedicine, the definition and therefore the moment of death is a matter of ongoing and disturbing dispute between two major channels of thought. Should we understand life to end at the point of cessation of cardiac function, or alternatively that of the brainstem? That whole logic is predicated, however, on the familiar binary of life/death that fails to address urgent concerns in three arenas: social-cultural imaginaries, postmodernist philosophy and increasingly exploratory bioscience. If there is always something about death that is uncanny, that exceeds rationalist thought, then we need to queer the concept and ask whether there are more sensitive ways of thinking the process of dying. The very concept of extended life for the recipient is no simple outcome, and the question of whose life has been prolonged is far from clear. My contribution touches on the idea of thinking transplantation in the mode of parasitism but will suggest an alternative Deleuzian way forward.

The field of human organ transplantation studies is shot through with questions concerning the concepts of selfhood and identity that continually reach out towards transmigration, displacement and haunting. Most particularly, heart transplantation is the site at which the parameters of human life and death are tested to their limits, not simply for the recipient but for the donor too. In conventional biomedicine, the definition and therefore the moment of death is a matter of ongoing and disturbing dispute between two major channels of thought. Should we understand life to end at the cessation of cardiac function, or alternatively that of the brainstem? The distinction is no trivial matter but is strongly tied to which utilitarian consequences are in play, and how interventions like the removal of organs for transplant should be operationalised. The whole issue is predicated, however, on the familiar Western binary of life/death that fails to address urgent concerns in three arenas: social-cultural imaginaries, postmodernist philosophy, and exploratory bioscience in which microchimerism defies a simple temporal cut. If there is always something about death that is uncanny, that exceeds rationalist thought, then we need to queer the concept and ask whether there are more sensitive ways of thinking the process of deceased donation. The very concept of extended life for the recipient is no simple outcome, and the question of whose life has been prolonged is far from clear. My approach touches on the idea of thinking transplantation in the mode of parasitism but will suggest an alternative Deleuzian way forward.

In the era of biopolitical thought and its concern with what constitutes the parameters of life, the emphasis has been firmly on the management of the living in every register of existence—social, political, ontological—without fully engaging with what, if anything, constitutes death as an irrecoverable endpoint. We understand that the dead still contribute as data as Puar (2010) points out, as environmental enrichment (The Corpse Project.), as the source of biological material through things like transplantation technologies (Sharp's 2006), or as the absent presence of memory, and so on, but what is rarely questioned is the putative break between the living and the dead. For all Western culture’s interest in zombies, ghosts, vampires and spectres, the intrigue of those uncanny modes arises from the supposition that they are quasi-animate forms that *should not* be living. Certainly life and death intrude on one another, but the prevailing psychosocial imaginary of the global North remains confident that the entanglement can be resolved into a familiar binary difference. What would it mean then to think not about death as a bounded category but as irreducibly entangled in the processes of life? Jacques Derrida is perhaps exemplary in his exhortation to live well with the dead and to accept the hauntological dimensions of all existence—by which he refers to those elements which are neither present nor absent, neither dead nor alive—while Gilles Deleuze provides a way of challenging the normative temporalities of death. I want to start, however, by looking at the implications of some more mundane considerations arising from the biomedical understanding of death and dying, particularly in relation to heart transplantation, and by reflecting on some empirical data as they illuminate my theorisation of the issues involved.

Over several years, I have collaborated on two major qualitative research projects in Canada: the *Process of Incorporating a Transplanted Heart* (PITH) initiated in 2007, which from 2011 segued into *The gift of life: A critical visual exploration of donor families’ responses to organ donation* (GOLA).1 Both projects entailed extended interviews with 25 and 22 respondents, respectively, all of which were both audio and video recorded. Regardless of the switch in focus from recipients to donor proxies, the question of death has been of crucial importance throughout, although in very different registers. For recipients faced otherwise with imminent demise, the death of the donor is a necessary facet of their own survival, and it produces, not surprisingly, a complex mix of relief, gratitude, obligation and guilt. Initially, many who have received a graft claim not to think about the provenance of the organ, but in interview the overwhelming majority express significant disturbance about their relation to the donor in which the other seems to persist within the self. It might be called a relationship of spectrality. The account of living on as a direct result of deceased donation brings life and death into an unwelcome focus that undermines the public interest in a heroic narrative in which both sides of the transaction can be lauded for their courage. When it comes to donor proxies (most often family members), the relation to life and death is even more complex, with their task of agreeing to the donation of specific organs falling into a tight window while their close relation is apparently still living. If death has already been declared it is generally too late to evacuate working organs, so biomedical staff are obliged to solicit consent to donation while at the same time exemplifying continuing care for the dying person. Once a medical determination has been made of brain death, life support is maintained, enabling the heart to continue beating until it has been removed and transported on ice to the site of implantation into another who is equally marked as dying. The process can be highly emotionally disturbing, but most biomedical professionals typically *display* no doubts about the moment at which ‘natural’ death has occurred and it would appear appropriate to terminate any life support technology. Donor families are far less certain and may feel acute distress, not only in being asked to rapidly consent during a time of shock and often disbelief, but also in frequently questioning if the person before them is really dead.

In all but certain unusual cases, the normative split between dead and alive prevails, and where donor families do refuse consent it is often for reasons other than a questioning of death itself. Yet there are some disturbing and highly visible anomalies that trouble the distinction. For example, even after the machinery that prolongs respiration and heart beat has been disconnected, a putatively brain dead body may show strong signs of animation, as in the so-called Lazarus sign (Taskin 2017). This often involves raising the arms over the head and then lowering them onto the chest, the biomedical explanation being that it is a spinal reflex arc that happens independently of the brain and has nothing to do with personhood. At this point, we might pause to wonder at the justification of excluding such phenomena which though rare in ‘death’ are an intrinsic part of everyday living. Indeed without such reflexes we would suffer all sorts of unnecessary trauma to our embodied selves, but by and large heart transplant teams are not phenomenologists but utilitarians. The connection between biological brain matter and personhood in the philosophy of consciousness is already contentious, but the overwhelming emphasis in the biomedical arena on the solid brain as the marker of proper life is a little puzzling. Neuroscientific research has revealed that the heart itself and its immediate surrounds are an area extremely rich in neuron activity that directly modulates the relation between emotion and cardiac function, and while the claim that the intracardiac nervous system constitutes a little ‘brain’ is deeply misleading, there is a clear indication that the heart is not simply controlled by the brain.2 In some invertebrates and particularly cephalopods, such as the octopus, there is effectively no central brain at all, but simply a dispersed network of neurons throughout the body. Yet the movement of an octopus cannot be reduced to non-intentional reflex insofar as the animal is thought to have the intelligence of a young human child, with a well-documented capacity for inductive learning and memory (Godfrey-Smith 2016). Evolutionary paths may be very different, but must we suppose that human reflex has no connection to such higher functions?

Even in the face of growing research, the implications of such considerations remain speculative, but my misgivings become clearer in the recent historical context of what constitutes death for a prospective heart donor. Up until the 1960s death was defined as the failure of the cardiorespiratory system, with the permanent cessation of breathing being the unquestioned mark of death. With the invention of the artificial ventilator, however, a dying patient could be resuscitated or stabilised so that the heart would go on beating for an indefinite period. Given that the 1960s were also the point at which organ transplantation became feasible, this technological intervention clearly created a conundrum in that it could no longer be certain that the patient had died. Partly in response to this, the biomedical definition of death—in Westernised societies at least—was shifted in 1967 to brain death which it was felt could be accurately assessed even though the body was warm, breathing and oxygenated.3 In other words, death was now synonymous to lack of brain function—which was taken to integrate the whole body—though there was never a clear explanation as to why the cessation of one organ should become the sole marker. As Peter Singer—an arch utilitarian who approved the choice—puts it, the new definition of death was ‘an ethical choice masquerading as a medical fact’ (Singer 1995, 50). What made it ethical for Singer was precisely the utilitarian calculus of weighing the prospect of other lives saved against the possible harm done to the donor. In short, the adoption of the brain dead criterion was always a fudge. In 2008 the US Presidents Commission revisited and amended the definition to claim that the destruction of the brain constituted death because it meant that the person could no longer engage in ‘commerce with the surrounding world’. What that phrase actually means is far from self-evident, and many families who go on supporting those on life support machines for months, even years, would claim that there *are* forms of rudimentary communication. A review by Shah, Kasper, and Miller (2015) of 43 geographically diverse research papers demonstrated that families, cross-culturally, ‘possess hope for a miracle or the belief that their family member may recover after brain death’ (293).4 The public discourse of biomedicine is not known for its embrace of indeterminacy, however, and such families are habitually characterised as deluded (Shah, Kasper, and Miller 2015). At root there may be a serious mismatch between the timelines of biomedical practices such as transplant surgery, which is strictly linear and teleological, and the lingering phenomenological temporality of life and death.5

That disparity underpins the immediate scenario of donation and retrieval of the organ where the codification of passing time is at its most acute and conflicted, as our qualitative study of 22 Canadian donor families shows. All consented to the donation of one or, more usually, several organs once brain dead had been established, yet the clinicians’ desire for a speedy procedure gave little time for bereaved relations to examine their own feelings and doubts. While none of our interviewees overtly questioned the distinction between cardiac and brain death, the sight of a still breathing, at least minimally reactive body was extremely disturbing for many. As Kathleen Fenton puts it, ‘society as a whole is not completely comfortable with the idea that a warm, pink patient is actually a corpse’ (Clarke, Fenton, and Sade 2016, 2056). At an everyday level, the concept of brain death is difficult to grasp—“I think he died in front of me,” said one mother, only to be told that her son had already been dead for several hours—and it may not be until the respirator is switched off that there is any final acceptance. What the lay donor families are unlikely to know is that new techniques—called heart beating transfer—now obviate the need to stop the heart at all. Once the heart can be removed from the ventilated donor, it is placed in a machine that perfuses it during transportation to the recipient’s location. In consequence the graft continues beating throughout, and in such cases cardiac death as such never occurs. For the clinical staff the ‘now’ of the life/death binary is what makes the whole procedure ethically acceptable, and it is possible that the new technique will disrupt that certainty once it becomes more widely used. For families, however, the anguish of uncertainty is already all too apparent.

All the donor proxies interviewed for the *GOLA* project—which was an art, social sciences, humanities and medical sciences collaboration—had agreed to donation whatever their misgivings. For just a few, it was a seamless transaction that scarcely raised any ontological anxieties and provoked only minimal speculation on the meaning and temporality of death. One robust 80-year-old retired professor (D20) who donated her husband’s organs told the interviewers, “I looked down at him and said that’s not my husband…the body is just cells…they’re not the person,” while the parents (D6) of a deceased daughter remarked, “We were a little bit on pins and needles to make sure it *[donation]* happened.” Most commonly, clinical staff were praised for their sensitive approach, but they could not, nevertheless, cover over the strangeness of the situation. As one mother (D18) puts it, they “were very professional and personal at the same time,” but “in the back of my mind I kept wondering was he really gone.” The majority of respondents, however, experienced the short process of gaining consent as highly disturbing—“like a pushy used car salesman,” said two bereaved sisters (D3) who had witnessed their “dead” brother’s legs still twitching and a tear falling from his face; or “like seagulls circling” as one bereaved husband (D1) described the clinical staff. A mother (D8) who still felt traumatised 36 months later told us that the time of her daughter dying was unbelievably bad because “she looked like she was just asleep,” so “I just wanted to punch them in the face…That’s your child being carved up to be doled out to other people.” Another mother (D16), this time of an adult son described 3 days of emotional turmoil when “you listen but you don’t hear…it’s gut wrenching.” Her profound doubts following consent pervaded her dreams: “I was having nightmares…when they were removing his heart and eyes, he was screaming.” Another (D2) said of the same period: “I felt some-one was surgically removing *my* heart.”

The provision of life support that is necessary during the quasi-death period to maintain the functionality of organs clearly creates an epistemological quagmire for those who are asked for consent, which does not end once the transplantations have been completed. This can be illustrated with reference to a particular case of a young First Nations man who had drowned but been temporarily revived. This was an especially lengthy and harrowing story in that the estranged non-indigenous mother (D14) was interviewed separately from her indigenous husband and daughter (D9). Everyone was in agreement that the deceased man, Eric,6 was highly spiritual, believed in saving others and would have wanted to donate his organs. The mother had pressed for donation right away and saw it as a gift received by recipients—“to be able to live on – that’s a huge gift”—rather than as a gift given by her son which would somehow enable *him* to live on *through* them. The father, however, had wanted to be sure and resisted consent, leading to an 8-day gap between the declaration of brain death and donation.

During that period he attended a drumming ceremony at the lake where Eric drowned, and felt that he was given certain signs from the natural world that convinced him that donation should go ahead: two loons on the lake that spoke to the calmness of Eric’s spirit, a crow that flew up overhead in response to a prayer for a sign from Mother Earth, and finally coming across a seemingly dead, but still sprouting tree which signalled the intertwining of life and death. For the father, living on did devolve on his son but in two conflicting ways. On the one hand his First Nations beliefs were opposed to donation because the body is cherished (as a gift in itself), the self is transcendent, and not least because Eric’s eyes were needed to show him the way to the spirit world. On the other hand he firmly believed that *both* Eric and the recipients would be enabled to live on through donation. Each parent and his sister talked about Eric actively saving five lives through the distribution of his organs and somewhat unusually they saw his continued being-in-the-world as a matter of everything being connected and sustained. The father’s raw grief at his loss was mediated by a sense of Eric’s continuing presence in his life and he stressed how he would like to ask the heart recipient if he ever wanted to ride a horse or climb mountains as his son did. For the family, Eric is both part of the recipients and may guide their behaviour, but he is also everywhere, beyond any specific temporal location. As the mother said about the interview, “I love talking about Eric…I hope he hears me,” while the father and the sister are certain they are still in communication. A spirit guide from another indigenous group had even contacted them with a message from Eric which they are inclined to accept at face value.

The specific indigenous imaginary7 at work here is clearly somewhat different from the Canadian mainstream and should remind us of the limitations of settler colonial epistemology and ontology,8 although intriguingly it also seems to speak to the kind of interconnections that Deleuze and Guattari (1987) call assemblages, of which more later. In that mode, dying is both a personal event, experienced within a conventional time frame, but also a continuing atemporal process that exceeds the binary of life/death. This is far from the contemporary operation of conventional biopolitics in which the threshold of mortality marks the cessation of being. Traditionally the disturbance of death is the endpoint of the social contract and of any further engagement with others. Such a highly rationalist and masculinist construction of the centrality of the living grounds, on the one hand, the biomedical impetus to preserve life at all costs and, on the other, a plethora of surveillance and control technologies that seek to establish and maintain the distinction between those deserving and undeserving of survival. I would contend that both branches are highly evident in the mode of organ donation, which above all is situated in the affluent technologised societies of the global North. The largely unseen apparatus of consigning and transporting viable organs is a highly complex logistical and biopolitical operation that in the end purports to rely solely on an apparently free choice—in most jurisdictions at least—to willingly donate body parts to unknown others. The whole emphasis of what are called OPOs (organ procurement organisations) is directed towards promoting the supposed altruism of donors and covering over any unsavoury mentions of death or dying. With heart donation, the donor is, of course, always dead, but that would hardly be apparent from the literature which stresses only the altruism of realising the gift of life. For all the smooth publicity, however, there is always a shortfall in organs offered and organs needed to meet the demand for heart transplantation to the extent that many countries, especially in Europe and including the UK, are turning to presumed consent where none has been given in advance. At the same time the illegal global trade in organs as a branch of neoliberal capitalism is thriving at the expense of those populations marked as undeserving. Throughout, disciplinary technologies mesh with the preservation of life and the denial of death.

What does all this mean to donor families reflecting on their decisions to assent to the transplantation process? The question of living on is central to their thinking, although the grief experienced may overwhelm other affects. The narrative favoured by OPOs clearly promotes the transplant organ as the so-called gift of life that offers the hope of survival to recipients, with the associated benefit to donor proxies that they have engaged in an altruistic act. There is recognition too that altruism is not entirely disinterested in that giving has therapeutic effects in introducing something positive into the scenario of loss. As one woman (D4) puts it, “I had to believe in something good. It was the most awful thing,” while an upbeat couple (D6) whose daughter died in a Road Traffic Accident stressed, “Do the recipients know how happy we are?” “Do they know we are on their team?” What is less acknowledged is that many proxies are more or less invested in the belief that through donation it is not only the recipient but also their dead loved one who will live on. It is certainly the case that donor proxies may express a deep concern for recipients and say that they would be devastated again should the recipient fail to flourish, but as our interviews revealed it is rarely that simple. As the father (D8) of young woman killed in a light aircraft crash told us, “we don’t need any more grief,” while his wife adds, “Ruth [the recipient] is a little piece of Lisa [their daughter]…if something happened to her, it would be another death for Lisa.” The empirical data show that the majority of donor families go on referring to transplanted hearts as still belonging to their own loved one, and as having an agency independent of the recipient, who in turn habitually experiences the transplanted organ as not fully incorporated to the self, but rather as a living reminder of the deceased donor. Typically as one respondent (D13) puts it, “There’s my sister’s heart beating away in someone else, bringing them joy,” and she thinks of the donated organs as “keeping them alive, that’s what they’re doing, they’re keeping others alive with their organs.” It is difficult to escape the conclusion that living on refers both to the survival of the recipient *and* to the donor. In some magical sense the necessary death of the donor is negated and she is not so much reincarnated in an other as she occupies a new location where aspects of the self can endure. As the mother (D2) of a teenage donor puts it, “I haven’t lost him – I know exactly where he is…he’s still giving,” or a daughter (D17) speaking of her father, “He lives on, he got to share himself with strangers.” There are perhaps some hints of parasitism in the strange alliance between the deceased donor and the recipient, where one of the organisms benefits at the expense of another, but given the marked emphasis on altruism it may better be thought of as a hauntological relation or symbiotic entanglement.

The whole procedure is greatly complicated where anonymity between recipients and proxies is enforced, although that requirement is breached with some frequency. In many jurisdictions—including in the geographical area of my own research—anonymity is legally mandated between transplant recipients and organ donors or their proxies. Any communication between donor families and heart recipients is vetted, depersonalised and anonymised prior to being shared, such that no one should know the identity of the giver and the receiver of ‘gifted’ organs. Despite the widespread terminology of the ‘gift of life’ to describe the transplanted organ, which implies an intimacy in the relation between the donor and the recipient, anonymity has been central to biomedical, psychosocial and societal practices and discourses around the process. The result is an inevitable disjunct between significant evidence that both individual recipients and donor families may be emotionally troubled by the prohibition, and the wider biopolitical context of transplantation procedures which operate through a systemic de-individualisation of donors and serve to endorse the commodification of their bodies. The practice of anonymous donation is deemed a public good—mediated by the biomedical community—in which the gift is not personal at all, but becomes an expression of community cohesion rather than an enactment between individuals. The bitter irony, nevertheless, is that those most intimately concerned are simultaneously encouraged to celebrate the individual ‘gift’ relationship and denied knowledge of their counterparts as either recipients or donors. Either way, anonymity intends to effect a profound depersonalisation that enables organs to be translocated in the service of utilitarian ethics. Although OPOs are not unaware of the strength of negative feeling about imposed anonymity, they go to some lengths to cover over identities, ostensibly for the protection of both donor proxies and recipients. There may, of course, some sense to this,9 but in general it is a highly paternalistic approach that over-rides the potentially mutual choices of those involved.

Throughout the pretransplant and post-transplant process, the donor is disembodied and effectively stripped of identity, seen only as the source of spare body parts precisely because in conventional biomedicine the heart is regarded merely as a pump, and transplantation as a technically mediated medical intervention with no metaphysical or ontological connotations.10 With regard to donor organs, the concept of commodification finds its justification in the tradition of mechanistic representation of the body in Western culture, and the emphasis on depersonalisation in the relatively recent procedure of heart transplantation evokes the Weberian concept of rationalisation and its principles of calculability, predictability and control. It precludes any consideration of the disturbances to embodiment and personal identity that arise from disturbances to the bodily morphology of an individual patient. In any case, the status of deceased donors—any ‘dead’ body really—is problematic in that they are both enduringly material—the source of further of life—and non-living, a spectral presence. The mechanistic model that informs the imposition of anonymity nevertheless over-rides the probable emotions of the organ donor proxies and recipients by sterilising the process through which they communicate. While such objectivity sometimes does serve a protective purpose in shielding both sides from unwanted expressions of intimacy, the bureaucratic operation of transplant support services dehumanises the participants and removes emotion from the logistical and biomedical procedures of procuring and transporting organs, matching donors and recipients on the basis of biometrics, and providing post-transplantation support. It is at times a deeply inappropriate and unwelcome scenario, given the deliberative disregard of human interaction. The relentless objectification of what is an inherently subjective and intimate process can be deeply damaging. Over 80% of our recipient cohort, for example, were reduced to tears of distress or despair in describing their struggles to write the anonymous letter implicitly demanded by their receiving the gift of life (Poole et al. 2011), while on the donor side there was similar anguish if nothing was heard from the anonymous recipients.

Right from the start with the original PITH project and subsequently with the GOLA research, our own study has been guided by a very different approach that initially referenced the philosophy of Merleau-Ponty (1968), for whom the body is never merely an object, and self-identity not given but constructed through embodied, spatial and temporal connections with others. As a phenomenologist, Merleau-Ponty describes the world itself in corporeal terms and individuals as participants in a network of inherently intersubjective encounters. Specifically, he understands the ‘flesh of the world’ as a living web of interconnections in which we are all implicated (Cataldi 1993). Subjective experience is to the fore, but Merleau-Ponty implies that the individual cannot be treated as an autonomous entity, but is always in process, in a state of becoming that relies both on the interface between the body and the world, and on the interactions between embodied selves. In place of the rigid and normatively framed autonomous self for whom the body is a possession that gives rise to property rights and questions of alienability, this kind of self is inseparable from, and only exists in virtue of, those who are others. The approach focuses not simply on the abstract interconnections between self and other, but more fundamentally on an intercorporeality in which bodies are woven together. In contrast to the impersonality of the rationalist approach, phenomenology deals in the felt experience of emotions and affects. More specifically, the literal replacement of the hearts of terminally ill patients with donor organs underscores their delicate intercorporeality, drawing together givers and receivers in an intimate relationship. Beyond the hard-edged rationalism of the clinical narrative, such an understanding of the embodied self lends credence to reports of complex feelings of connection—even kinship—of heart transplant recipients with their donors. That such feelings can operate even under conditions of personal anonymity surely demonstrates their potency. Nonetheless, the pragmatic procedures of organ transplantation rarely take the lived experiences of either recipients and donors (or their proxies) into account. Beyond the insistent gift of life rhetoric, the biomedical model of organ procurement, donation and transplantation encourages recipients to fragment the donor’s body and objectify organs as replaceable mechanical parts. Ideally the deceased donor’s once-personalised self would be disavowed.

What is really at stake in this seemingly uncaring approach opens up another dimension of anonymity in organ procurement and donation that I will touch on just briefly. While it is ethically routine to decry the international trade in scarce organs, the commodification of the body parts that it evokes is not confined to illicit activity. It is fundamental to the operation of transplantation as both enhancing life and seeking death. The thanatopolitics of transplantation is not simply an aberrant side effect. Once the human body is thought of as an object of commodification in the Marxist sense, its specific fragmentation in the transplant scenario ultimately renders the organs of the deceased donor as objects, with a use-value, shaped by quality, quantity and utility, and determined through consumption. Evoking commodity fetishism, Sharp's (2006) compelling interpretation of the anonymous process of organ procurement and transplantation exposes its reliance on elaborate forms of metaphorical thinking, on the part of all those involved, that obscure the origins of displaced body parts. It is above all a process of mystification, as can be clearly seen in the contradictory biomedical discourse that strongly encourages the recipient to be grateful to the donor family for the gift of life while at the same time depersonalising the donor as merely the source of transferable spare parts. For biomedical professionals, the suspension of personhood at the moment of brain death marks the moment at which the body becomes ‘a reserve of commodities’ that will swiftly circulate in the transplant economy. Yet as Lindberg (2013) notes, ‘the imperceptible transformation of gratuitous organs into precious commodities is one of the big taboos of the transplantation medicine’ (2013, 252). Such biopolitical concerns are seemingly far removed from the troubled register of personal exchanges, but in the end they interlock. Under the rubric of transplantation, the once identifiable human flesh of the donor vacates the space of the intimate and familial and becomes the object of public utility and technological expertise. The sense of an individual death is covered over, and those left behind are denied the physicality of an integral body to mourn, although communication between recipients and proxies may imaginatively seek to reconceive that lost personhood. Recipients, in particular, *are* strongly encouraged to write anonymised letters, with at least an expression of gratitude, although donor proxies—longing for some indication of the location of the gifted organ—may initiate the correspondence. Given that any identifying text—gender, ethnicity, names of pets, age, employment categories—is redacted at source, it is hardly surprising that the quasi-obligation to engage in a wholly anonymised exchange does not often provide the resolution that is sought, but becomes a focal point of overt disturbance for givers and receivers alike.

In the context of anonymity, the gift rhetoric that is the common currency of any communication unintentionally flirts with danger by encouraging donor proxies to look for a return on their supposed altruism when nothing substantial can be offered. At an unproblematised level, the implicit demand for reciprocity can collapse into ‘the tyranny of the gift’ for both parties.11 Moreover, the exchange model of the gift, originally associated with the anthropological work of Mauss (1990),12 suggests that any donation exceeds its mere materiality to figure something intrinsic to the giver. As Mauss writes, ‘one gives away what is in reality a part of one’s nature and substance’ (1990, 10), and that ‘the objects are never completely separated from the men who exchange them’ (1990, 31). In line with the instinctive feelings of proxies, this suggests that something of the donor does indeed live on in another, as is sometimes explicitly acknowledged by recipients themselves. More often, however, the uncanny persistence of the other manifests as a disconcerting awareness that one’s own embodied being is now hybrid. The same impressions are strongly reflected in the popular imagination, where representations of transplantation abound with uneasy narratives that express an underlying fear that the personal characteristics of the deceased donor might take possession of the recipient, or that s/he might reappear as a spectral presence. It is not just—as phenomenology might predict—a change that could be assimilated in the fashioning of a new embodied self, but of a self that is haunted by irregular traces of otherness. For Derrida (1994), the coming of the other is inevitable and it always constitutes a hauntological relationship between absence and presence, life and death, as well as self and other, the very issues that frame the existential register of heart transplantation.13 Although the model proposed by Mauss might appear at odds with the very different understanding of the gift relation offered by Derrida (1992), which does not rely on exchange and where identity *should not* be known, there is in both a hauntological dimension that finds resonance in the concept of ‘living on’ that is the counterpart of deceased donation.

All forms of organ and tissue donation might be expected to evoke the absent presence of the other, but throughout our interview material, which often touches on other forms of donation, it is invariably the heart, with all its cultural baggage, that is the centre of attention. All the respondents spoke of donors who were the source of multiple organ and tissue transplants, but no other organ had the power to disturb the normative teleology of life and death, that was attributed to the heart. One bereaved mother (D16) expressed something of this breakdown: “I miss him, I miss him, I carry him in my heart for ever. You carry him for 9 months, to grow up, to have a normal progression,” and unusually she saw the trope of ‘living on’ as extending to herself. Imagining what she would tell the recipient, she says, “Take care of the heart and it will look after you for the rest of your life,” adding “I hope he’d look after his heart. After all I created that heart…(I hope) he’s a good man.” In that context, it is no surprise that the death of a recipient—if it is known—can come as another personally felt death. As one mother told us, “We need to know that they lived.” A single respondent (D12) cited the spare parts model of transplantation—“it’s like taking an engine out of a car”—and a couple more referred to the heart as a pump, but for the majority there is nothing impersonal about the transfer. Against the clinical metaphor of replaceable machine parts, the counternarratives of the gift of life and donor altruism promoted by OPOs are the ones that make most intuitive sense to donor families. Where for the clinical professionals the success or failure of the procedure is a matter of objective biomedical measures, the longer term emotional impact of the decision on the lives of donor families and recipients alike tells another story in which the clear distinction between life and death is lost.

With regard to living on, however, the approved notion is supposed to be purely symbolic, or at least referencing only the recovering recipient. The authorised discourses stop short at a template of a dying, and then deceased, donor superseded by the putatively restored life proper to the recipient alone. Despite the keen beliefs of donor proxies, indicated in the quotes above, there is nothing in official models to disturb the succession of singular selves. In the biomedical imaginary—which constrains but does not wholly suppress the intimations of donor families—if only inanimate material is transferred then no ontological anxieties should arise. At present, the imposition of anonymity matters both positively and, more often, negatively precisely because transplantation *is* seen as an exchange that grounds an intimate and ongoing relation between self and other. Yet, as Davies (2006, 8) notes in her research on kidney donation, ‘attempts by either donor or recipient to construct a social relationship is viewed by professionals as pathological, something to be treated through counselling’ (2006, 260). What is really needed is a radical shake-up of a realm where personal identities are the privileged markers of living, and dying, that instead moves towards a biopolitics of transplantation that emerges from postmodernist insights into multiplicity, fluidity and vitalism. The theoretical models that I favour require a wholesale turning away from the central tenets of the Western imaginary, not least concerning the integrity of bodies, the sovereignty of the self, the meaning of death and the justice of exchange. Above all, if there is always something about death that is uncanny, that exceeds rationalist thought, then we need to queer the concept and ask whether there are more perceptive ways of thinking the process of dying.

Before pursuing that different theoretical—and in the end more affective approach—I want to quickly mark that the biology of transplantation is far less straightforward than it purports to be. I have already outlined the difficulty of making a biomedically coherent cut between life and death once a donor body is maintained in a perfused state and the ontological anxiety that invokes in donor proxies, but there is an even more complicating factor. The phenomenon of microchimerism in which a single body may host two or more entirely separate sets of DNA has long been acknowledged as a feature of maternal-fetal interchange in pregnancy and subsequently, and more controversially, since the early 1990s as a potential outcome of transplantation (Starzl et al. 1992; Quaini et al. 2002). When a heart or indeed any other organ or tissue is grafted into a recipient, it carries with it the DNA coding and the unique immune system markers (human leucocyte antigen, HLA) of the donor.14 Unlike other forms of hybridity where merging within each cell may occur, the distinctive cell line of the donor is not assimilated but persists in parallel to that of the recipient. Until a couple of decades ago, it was believed—if the phenomenon was considered at all—that the non-self coding would remain in situ at the location of the transplant, or that if any circulation occurred it would be of just a few weeks duration until the self cells re-established themselves throughout the body. Research now indicates that non-self cells circulate widely and are transported not only in the blood but can migrate and accumulate in organs and tissues other than the one transplanted. Moreover, it has been repeatedly shown that non-self cells persist for decades.15 An autopsy of a female heart recipient with a male donor, for example, could reveal male (Y-marked cells) in the brain or liver.

At this point in time, the existence and persistence of microchimerism are no longer doubted, but its cause—transplantation is just one of several possible sources—and its effect remain highly disputed in the bioscience of immunology. The major controversy revolves around the question of whether it should be regarded as pathological or beneficial, or perhaps both in different contexts. Biomedicine aside, the implications of microchimerism have become an arena of deep fascination for biophilosophy, particularly with reference to the notion of immunity, but also for the instability it brings to questions of self and other and life and death (Pradeu 2012; Staikou 2014; Shildrick 2019). If microchimerism is an almost certain outcome of transplantation, then it would appear that the intuitive feelings that constitute what I am calling a hauntology have a surprising biological endorsement. Once a donor’s DNA and HLA are embodied by the recipient but remain unassimilated, then self-identity is no longer certain and the trope of living on in another begins to make material sense. Microchimerism is as yet a little known aspect of transplantation—medicine like other epistemological systems tends to work in silos where cardiologists may be unaware of the work of immunologists, for example—and it is unlikely that the overwhelming majority of lay people involved have any knowledge of its operation. But just as the public understanding of science has very swiftly encompassed the existence of the human microbiome, although its ontological implications have yet to be grasped, so too microchimerism seems poised to give weight to feelings that have hitherto appeared to be merely speculative and even somewhat discreditable. It is not that bioscience is the final arbiter of the truth of the body, or even that there is any fixed point of truth, but that the question of intracorporeality that microchimerism introduces may give credence to the sense of a hauntological relationship between the recipient and donor in transplantation.

As a philosopher concerned with experiential states, I have long felt highly ambivalent about organ transplantation, initially from witnessing the ongoing disruption of recipients (which I have scarcely touched on here16), but more recently in response to proxy donors and their distress around the ambiguities of life and death. Fear of death is ubiquitous both for its putative termination of the singular self, and paradoxically because the dead have haunted the imaginaries of every age and culture. The first consideration is easy to understand and hard to shake, but with respect to transplantation the second—on both sides of the transfer—has the capacity to evoke the negative spectre of parasitism where one lives on at the expense of the other, but also the creativity of assemblage—the coming together of disparate elements—and sustainability. I want to end, then, by picking up a final quote from the interview with a female professor (D21). Despite her cool rationalism, she said something of her husband’s death that can move us on, “It’s no longer life, it’s potential,” and perhaps she was intuitively echoing the insight of Deleuze (1990), for whom the ontology of death cannot be limited to its manifestation in the sovereign subject, but is ‘incorporeal and infinitive, impersonal’ (1990, 151), beyond the concerns of any individual. In parallel, human life itself is not a finite essence, actualised in the limited lifespan of an individual, but rather a form that is temporally and spatially expansive, a component of the enveloping cycle of becoming that comprises all types of living beings, organisms, as well, in Deleuzian terms, as machines. Although each individually identified human life *is* marked by discrete episodes such as pregnancy, transplantation or dying that effect radical changes or transformations for that specific person, in another sense, those modes also transcend any singular form of embodiment and can be understood as intangible and atemporal forces and points of intensity. The Deleuzian approach rejects the modernist and very Western imaginary of an atomistic and sovereign subject, and celebrates instead not sustained and independent ‘being’—the enduring sense of self—but a material and processual state of becoming in which any *individuality* is provisional and always in the course of unravelling (Deleuze and Guattari 1987). In short, the boundaries of the selfsame are superseded by the macro-context of collective becoming.

As Deleuze sets out, each of us is entangled in what he calls assemblages, those fields of energy reliant on multiple and shifting networks of interconnections, both organic and inorganic, that constitute life itself. In such heterogeneous orders, what matters are the mutual interactions and the irreducible hybridity of form that exceeds the unique experiences and singular embodiment of the individual (Shildrick 2015b). In place of existing epistemologies predefining and limiting the possible connections open to the singular self, in an assemblage, the dynamic is reversed with the interconnections themselves generating meaning. That dynamic better enables us to understand what is at stake in chimerism, where disparate cell lines continue to circulate in conjunction, mutually affective and functioning through a new mode of configuration rather than being assimilated as such. Where the metaphor of parasitism may at first appear to have some purchase in the mode of living on after organ transplantation,17 its evocation of self/other antagonism has little in common with the Deleuzian model of assemblage. That postbinary approach insists that human life is always inherently entangled not only with other living beings but with a plethora of more or less animate technologies and processes. In blurring the boundaries of otherness, entanglement conjures up neither parasitism nor the absence/presence of Derridean hauntology where an ethical relation must exist. Assemblage theory establishes the productive capacity of connectivity and its incessant transformation, and may offer a template for rethinking organ transplantation, not as a one-off event, but as an *ongoing* and fluid project that encompasses both the recipient and the deceased donor.

The medical humanities to date have a limited appreciation of the Deleuzian intervention into modernist ontology and epistemology, but assemblages are highly significant in enabling us to reconsider the nature of embodiment, not least in transplantation (Shildrick 2015a). Deleuze reminds us, moreover, that what is at issue is not functional efficacy, or the expectation that a singular life will prevail. In the liberal humanist context in which only individual selfhood counts, it is understandable that donor families wish to see the donor living on in another, and that recipients should experience disturbance when they feel themselves no longer the self who preceded the surgery. In the Deleuzian mode, however, life is not the possession of an individual but an intense process of becoming other that does not meet a limit in death. In that sense, the deceased donor—whose varying organs and tissues will typically be distributed among multiple recipients—continues to contribute to the ongoing flux and flow of life. Living on impacts many bodies who are thus brought into relation with one another. With regard to the survival of the transplant recipients, what matters in Deleuzian terms is neither guilt about the donor’s death nor any obligation to bear witness to them, but the capacity to affirm life, both in its renewed potential and in its endurance. The good life is one that overflows individual boundaries and transforms itself even in the face of adversity, moving always towards new possibilities of becoming other than itself. In an important sense, the death of the donor is negated, but not by the transfer of personal characteristics to another. It is an impersonal relation in which both the giver and the receiver live on in a new and sometimes volatile assemblage.

At the present time, the sociocultural imaginary of the global North remains dominated by quasi-Cartesian conceptions of the body, and by the separation of self and other, both modes that facilitate the surgical onslaught of heart transplantation through the machine model and at the same time generate deep anxieties when that model fails to correspond with lived experience. The research I have outlined attempts to think beyond transplantation as a heroic intervention that defies death, and to recognise that organ donation generates a disturbing and potentially painful awareness that the boundaries of the self are no longer certain. It suggests two new registers of thought: first, if the personal event of dying were seen simultaneously as the recomposition of life under new relations of sustainability, then mortality itself would not be a failure; and second we might reimagine existence not in terms of a recovered self, but through dynamic incorporeal coexistence and forms of assemblage. In contradistinction to modernist societies that regulate what is deemed appropriate to any given body, the Deleuzian approach advocates pushing to the limits of what is possible, embracing uncertainty and radical change, and sustaining becoming, however that plays out. The task is surely to begin to change the sociocultural imaginary. One step might be to push the authoritative discourse of biomedicine to give up its investment in mastery and chrononormativity and openly explore what lies beyond the wound of the sutured body. It requires a paradigm shift on the part not just of clinicians, but all of us, to bring the threads together. Revisiting the case of the First Nations family, it is apparent that beyond the Western mindset there are already more positive ways of thinking death that can speak to a wider atemporal vitalism. We cannot deny the immediate pain or grief of individual demise, but it can be mitigated through an open encounter with another dimension where living and dying, self and other, absence and presence are irreducibly entangled.

## Data Availability

Data sharing not applicable as no datasets generated and/or analysed for this study.

## Notes

1. The original team consisted of Heather Ross (cardiologist), Pat McKeever and Jen Poole (health sociologists), Susan Abbey (psychiatrist), Margrit Shildrick (philosopher), Enza DeLuca and Oliver Mauthner (nurse-trained interviewers), and Alexa Wright, Andrew Carnie, Ingrid Bachmann and Catherine Richards (artists). Full ethical approval under REB #07-0822-BE.

2. See Campos et al. (2018). Research is focused on animal studies, but the implications for human physiology cannot be overlooked.

3. In recent years, donation after circulatory death (DCD) has been reinstated as an acceptable procedure. Donors may be those who do not fulfil brain death criteria but have no hope of recovery and for whom the withdrawal of life support is in their supposed best interests (Manara, Murphy, and O’Callaghan 2012). In 2016, the British Transplantation Society recommended against DCD for heart transplants, but restricted—and mostly paediatric—procedures are underway in many jurisdictions, including the UK, USA and Canada.

4. For Agamben (1998), the brain-dead body is a paradigm case of bare life and therefore open to sacrifice, but for those at the bedside, hopes of recovery—or perhaps just endurance—sustain the sense, not of death, but of the extreme vulnerability of a still embodied self.

5. See Lock (2002) for an analysis of the criteria and meaning of death in contrasting North American and Japanese contexts.

6. All names have been changed.

7. Indigeneity is a somewhat uncomfortable blanket term naming a typically Western form of categorisation. In Canada, indigenous peoples may identify with the very different cultures of Métis, Inuit or First Nations, none of which should be seen as uniform in themselves. To speak of an indigenous imaginary implies only alternative ways of thinking unfamiliar to more common Canadian mores.

8. There are relatively few studies of organ transplantation within indigenous populations, although the work of Webb and Shaw (2011) with Maori communities is an exception.

9. The claim to kinship between donor families and heart recipients, for example, may be deeply unwelcome (Dicks et al. 2018).

10. Fox and Swazey (1992) were among the first to offer a critique of the ‘spare parts’ model from the perspective of the humanities.

11. The term was first coined by Fox and Swazey (1992) in relation to a range of organ transplantation procedures.

12. The exchange itself is theoretically endless in the Maussian model, with each party consecutively occupying the position of the giver or the receiver. In the context of heart transplantation, the initial sending of a letter—even under the strictures of anonymity—implicitly demands a reply, which in turn necessitates its own reply and so on.

13. Derrida (1994) *Specters of Marx: The State of the Debt, the Work of Mourning and the New International*, trans. Peggy Kamuf. New York: Routledge.

14. An individual’s immune system is characterised by human leucocyte antigens (HLA) expressed by a group of genes on a single chromosome. There is rarely a complete match of HLA between two individuals, except in the case of monozygotic twins. Given that heart transplantation relies on deceased donation, there is effectively never a match.

15. I have already written extensively about the research behind these claims. See Shildrick (2016), Shildrick (2017), 2020.

16. See Ross et al. (2010) for an analysis of the early data from the *Process of Incorporating a Transplanted Heart* project.

17. Nancy (2002) text *L’Intrus*, written after his own heart transplant, initially suggests a form of parasitism, although it moves to a more Derridean conclusion: ‘The *intrus* is no other than me, my self’ (13).

## References

[R1] Agamben G. 1998. Homo Sacer. Sovereign Power and Bare Life. Translated by Daniel Heller-Roazen. Stanford: Stanford University Press.

[R2] Campos I. D. , Pinto V. , Sousa N. , and Pereira V. H. . 2018. “A Brain within the Heart: A Review on the Intracardiac Nervous System.” Journal of Molecular and Cellular Cardiology 119: 1–9. 10.1016/j.yjmcc.2018.04.005. 29653111

[R3] Cataldi S. 1993. Emotion, Depth, and Flesh: A Study of Sensitive Space: Reflections on Merleau-Ponty’s Philosophy of Embodiment. New York: SUNY Press.

[R4] Clarke M. J. , Fenton K. N. , and Sade R. M. . 2016. “Does Declaration of Brain Death Serve the Best Interest of Organ Donors Rather than Merely Facilitating Organ Transplantation?” The Annals of Thoracic Surgery 101 (6): 2053–58. 10.1016/j.athoracsur.2016.01.100. 27112652

[R33] Davies G. 2006. "Patterning the Geographies of Organ Transplantation: Corporeality, Generosity and Justice." Paper Revised for the Transactions of the Institute of British Geographers. http://eprints.ucl.ac.uk/10968/1/10968.pdf.

[R5] Deleuze G. 1990. The Logic of Sense. Translated by M. Lester. London: Athlone Press.

[R6] Deleuze G. , and Guattari F. . 1987. A Thousand Plateaus. Translated by Brian Massumi. Minneapolis: University of Minnesota Press.

[R7] Derrida J. 1992. Given Time: 1. Counterfeit Money. Translated by Peggy Kamuf. Chicago: University of Chicago Press.

[R32] Derrida J. 1994. Specters of Marx: The State of the Debt, the Work of Mourning and the New International. Translated and edited by Kamuf Peggy . New York: Routledge.

[R9] Dicks S. G. , Northam H. , van Haren F. M. P. , Boer D. P. , and Dicks S. . 2018. “An Exploration of the Relationship between Families of Deceased Organ Donors and Transplant Recipients: A Systematic Review and Qualitative Synthesis.” Health Psychology Open 5 (1): 2055102918782172. 10.1177/2055102918782172. 30083368 PMC6069040

[R10] Fox R. C. , and Swazey J. P. . 1992. Spare Parts: Organ Replacement in American Society. New York: Oxford University Press.

[R11] Godfrey-Smith P. 2016. Other Minds. the Octopus, the Sea, and the Deep Origins of Consciousness. New York: Farrar, Straus and Giroux.

[R12] Lindberg S. 2013. “The Obligatory Gift of Organ Transplants: The Case of the Finnish Law on the Medical Use of Human Organs, Tissues, and Cells.” Alternatives: Global, Local, Political 38 (3): 245–55.

[R13] Lock M. 2002. Twice Dead. Organ Transplants and the Reinvention of Death. Berkeley: University of California Press.

[R14] Manara A. R. , Murphy P. G. , and O’Callaghan G. . 2012. “Donation after Circulatory Death.” British Journal of Anaesthesia 108: i108–21. 10.1093/bja/aer357. 22194426

[R15] Mauss M. 1990. The Gift: The Form and Reason for Exchange in Archaic Societies. Translated by W. D. Halls. New York: W.W. Norton.

[R16] Merleau-Ponty M. 1968. The Visible and the Invisible. Evanston: Northwestern University Press.

[R17] Nancy J.-L. 2002. L’Intrus. Translated by Susan Hanson. Ann Arbor: Michigan State University Press.

[R18] Poole J. M. , Shildrick M. , De Luca E. , Abbey S. E. , Mauthner O. E. , McKeever P. D. , and Ross H. J. . 2011. “The Obligation to Say ‘Thank You’: Heart Transplant Recipients’ Experience of Writing to the Donor Family.” American Journal of Transplantation 11 (3): 619–22. 10.1111/j.1600-6143.2010.03419.x. 21342451

[R19] Pradeu T. 2012. The Limits of the Self: Immunology and Biological Identity. Translated by Elizabeth Vitanza. Oxford: Oxford University Press.

[R20] Puar J. K. 2010. “Prognosis Time: Towards a Geopolitics of Affect, Debility and Capacity.” Women & Performance 19 (2): 161–72. 10.1080/07407700903034147.

[R21] Quaini F. , Urbanek K. , Beltrami A. P. , Finato N. , Beltrami C. A. , Nadal-Ginard B. , Kajstura J. , Leri A. , and Anversa P. . 2002. “Chimerism of the Transplanted Heart.” New England Journal of Medicine 346 (1): 5–15. 10.1056/NEJMoa012081. 11777997

[R22] Ross H. , Abbey S. , De Luca E. , Mauthner O. , McKeever P. , Shildrick M. , and Poole J. . 2010. “What They Say versus What We See: ‘Hidden’ Distress and Impaired Quality of Life in Heart Transplant Recipients.” The Journal of Heart and Lung Transplantation 29 (10): 1142–49. 10.1016/j.healun.2010.05.009. 20580266

[R23] Shah S. K. , Kasper K. , and Miller F. G. . 2015. “A Narrative Review of the Empirical Evidence on Public Attitudes on Brain Death and Vital Organ Transplantation: The Need for Better Data to Inform Policy.” Journal of Medical Ethics 41 (4): 291–96. 10.1136/medethics-2013-101930. 24769621

[R24] Sharp L. 2006. Strange Harvest: Organ Transplants, Denatured Bodies, and the Transformed Self. Berkeley: University of California Press.

[R26] Shildrick M. 2015. “Staying Alive: Affect, Identity and Anxiety in Organ Transplantation.” Body & Society 21 (3): 20–41. 10.1016/0140-6736(92)91840-5. 1351558 PMC2950640

[R30] Shildrick M. 2015. “‘Why Should Our Bodies End at the Skin?’: Embodiment, Boundaries, and Somatechnics.” Hypatia 30 (1): 13–29. 10.1111/hypa.12114.

[R38] Shildrick M. 2016. “Chimerism and immunitas: The Emergence of a Posthumanist Biophilosophy.” In Resisting Biopolitics: Philosophical, Political and Performative Strategies, edited by Wilmer S. and Zukauskaite A. , 95–108. London: Routledge.

[R37] Shildrick M. 2017. “Individuality, Identity and Supplementarity in Transcorporeal Embodiment.” In Finite but Unbounded: New Essays in Philosophical Anthropology, edited by Cahill Kevin , Gustafsson Martin , and Wentzer Thomas Schwarz , 153–72. Berlin: De Gruyter.

[R35] Shildrick M. 2019. “(Micro)Chimerism, Immunity and Temporality: Rethinking the Ecology of Life and Death.” Australian Feminist Studies 34 (99): 10–24. 10.1080/08164649.2019.1611527.

[R36] Shildrick M. 2020. “Matters of the Heart: Temporality and Microchimeric Entanglements.” In Entangled Bodies: Art, Identity, Intercorporeality, edited by El Sheikh Tammer , 59–79. Wimington, DW: Vernon Press.

[R25] Singer P. 1995. Rethinking Life and Death. Oxford: Oxford University Press.

[R34] Staikou E. 2014. “Putting in the Graft: Philosophy and Immunology.” Derrida Today 7 (2): 155–79. 10.3366/drt.2014.0087.

[R27] Taskin Ş. K. 2017. “Spinal Reflexes in Brain Death Cases.” Transplantation 101: S132.

[R28] The Corpse Project . n.d. “The Science of Decay and Decomposition.” http://www.thecorpseproject.net/decompositionscience/.

[R29] Webb R. , and Shaw R. M. . 2011. “Whanau, whakapapa and Identity in Experiences of Organ Donation and Transplantation.” Sites: A Journal of Social Anthropology and Cultural Studies 8 (1): 40–58. 10.11157/sites-vol8iss1id154.

